# The validity of diagnostic cut-offs for commercial and in-house scrub typhus IgM and IgG ELISAs: A review of the evidence

**DOI:** 10.1371/journal.pntd.0007158

**Published:** 2019-02-04

**Authors:** Kartika Saraswati, Meghna Phanichkrivalkosil, Nicholas P. J. Day, Stuart D. Blacksell

**Affiliations:** 1 Mahidol-Oxford Tropical Medicine Research Unit, Faculty of Tropical Medicine, Mahidol University, Bangkok, Thailand; 2 Centre for Tropical Medicine and Global Health, Nuffield Department of Medicine, University of Oxford, Oxford, United Kingdom; 3 Eijkman-Oxford Clinical Research Unit, Eijkman Institute for Molecular Biology, Jakarta, Indonesia; University of Tennessee, UNITED STATES

## Abstract

**Background:**

Scrub typhus is a neglected tropical disease that causes acute febrile illness. Diagnosis is made based upon serology, or detection of the causative agent–*Orientia tsutsugamushi–*using PCR or *in vitro* isolation. The enzyme-linked immunosorbent assay (ELISA) is an objective and reproducible means of detecting IgM or IgG antibodies. However, lack of standardization in ELISA methodology, as well as in the choice of reference test with which the ELISA is compared, calls into question the validity of cut-offs used in diagnostic accuracy studies and observational studies.

**Methodology/Principal findings:**

A PubMed search and manual screening of reference lists identified 46 studies that used ELISA antibody cut-offs to diagnose scrub typhus patients, 22 of which were diagnostic accuracy studies. Overall, 22 studies (47.8%) provided little to no explanation as to how the ELISA cut-off was derived, and 7 studies (15.2%) did not even state the cut-off used. Variation was seen locally in reference standards used, in terms of both the diagnostic test and cut-off titer. Furthermore, with the exception of studies using ELISAs manufactured by InBios, there was no standardization of the selection of antigenic strains. As a result, no consensus was found for determining a cut-off, ELISA methodology, or for a single value diagnostic cut-off.

**Conclusions/Significance:**

We have concluded that there is a lack of consensus in the determination of a cut-off. We recommend interpreting the results from these studies with caution. Further studies will need to be performed at each geographic location to determine region-specific cut-offs, taking into consideration background antibody levels to discriminate true disease from healthy individuals.

## Author summary

Scrub typhus is a neglected tropical disease that causes acute fever and can cause serious complications without appropriate antibiotic treatment. Diagnosis is usually made by the detection of specific antibodies or the causative agent–*Orientia tsutsugamushi*. Specific antibodies can be detected using ELISA technology however there is an apparent lack of standardization in the development of cut-offs used in diagnostic accuracy studies and observational studies. This study assessed 46 studies that used ELISA antibody cut-offs to diagnose scrub typhus patients. Overall, 22 studies (47.8%) provided little to no explanation as to how the ELISA cut-off was derived, and 7 studies (15.2%) did not even state the cut-off used. Furthermore, with the exception of studies using ELISAs manufactured by InBios company, there was no standard approach to the selection of antigenic strains, and therefore they may not be representative of the local antigenic strains causing disease. As a result, we have concluded that there is a lack of consensus in the determination of a cut-off. We recommend interpreting the results from these studies with caution and further studies will need to be conducted at each geographic location to determine region-specific cut-offs take into consideration background antibody levels to discriminate true disease from healthy individuals.

## Introduction

Scrub typhus is a neglected tropical disease caused by the obligate intracellular bacterium *Orientia tsutsugamushi* [1]. Transmission of the bacteria to humans occurs via the bite of larval trombiculid mites, known commonly as chiggers [2]. It was formerly thought to be confined to the ‘tsutsugamushi triangle’, encompassing Pakistan, Northern Australia and parts of Russia. However, cases acquired in Chile [3, 4], possibly Africa [5, 6], as well as the Middle East [7] (by a proposed novel species *O*. *chuto*), have been reported, suggesting that its endemicity may be more widespread than previously thought.

Patients typically present with acute febrile illness, but if left untreated, this may progress to systemic infection and multi-organ failure, contributing to an estimated median mortality rate of 6.0% for untreated and 1.4% for treated scrub typhus [8] highlighting the importance of early and accurate diagnosis. A characteristic necrotic lesion, or eschar, at the inoculation site may serve as a diagnostic clue, however its presence varies, ranging from 9%-97% depending on the population [9, 10]. Given that other febrile illnesses such as typhoid, dengue and leptospirosis have similar clinical manifestations as scrub typhus, laboratory test is essential to differentiate scrub typhus from other undifferentiated fever [11].

Serological methods are more often used to diagnose scrub typhus due to their simplicity and cost-effectiveness [12]. The indirect immunofluorescence assay (IFA) is considered the “gold standard,” but the requirement of a fluorescent microscope and the subjective nature of reading slides limits its application in rural areas where this disease is most prevalent [8, 11–14]. The Weil-Felix test is convenient to perform but suffers from poor sensitivity and specificity [13, 14]. Given the limitations of other serological methods, the enzyme-linked immunosorbent assay (ELISA) is acknowledged as a reasonably simple to perform alternative, providing an objective optical density (OD) result using an automated plate reader, that is reproducible in most clinical laboratory settings [13].

Despite the apparently standardized and objective ELISA platform, the diagnostic accuracy is influenced by methodological and patient factors. Methodological factors may include the composition of antigenic strains and their origin, and the choice of diagnostic cut-off. Patient factors are mainly centered on elevated levels of background immunity in endemic areas that may give rise to false positive results. Therefore, to ensure accuracy of diagnosis, standardized methodologies and locally validated OD cut-off levels for ELISA are urgently needed [8].

This review therefore aims to summarize (1) the differences in ELISA methodologies, (2) the OD cut-offs used for diagnosing scrub typhus in research, and (3) the rationale behind the selection of certain OD cut-offs for scrub typhus diagnosis in previously published diagnostic accuracy studies and observational studies.

## Materials and methods

### Search strategy and eligibility criteria

A scoping review was performed. Searches were performed by one author (MP) on the PubMed electronic database using the following search terms: “scrub typhus,” “tsutsugamushi”, “immunoassay”, and “ELISA”. The search was restricted to papers published in English, up to 16^th^ October 2017. The titles and abstracts were screened for relevance. The full-text of relevant articles were assessed to determine eligibility. Diagnostic accuracy and observational studies using ELISA to diagnose scrub typhus in human were included. We excluded co-infection studies, case studies and studies investigating variations of the conventional ELISA methods (e.g., dot-ELISA). Reference lists of the relevant articles were also screened in order to identify additional studies. The protocol of this review was registered in the International Prospective Register for Systematic Review (PROSPERO) with registration number CRD42017078596.

### Data extraction and analysis

Data was extracted by one author (MP), and where the information was unclear a second researcher was consulted (SDB). Details of the sample size, location, study date, reference test, cut-off, method used to calculate the cut-off, and ELISA methodology (antigenic strain and antibody isotype) were compiled into summary tables. The studies were grouped according to study design (diagnostic accuracy study or observational study), type of ELISA (in-house or commercial), and study location. The data was summarized using narrative synthesis. We did not evaluate minutiae of individual ELISA protocols, but instead focusing on the wider issues such as the methodologies used to determined diagnostic cut-offs.

## Results

### Summary of studies

#### Study types

**Fig 1:**
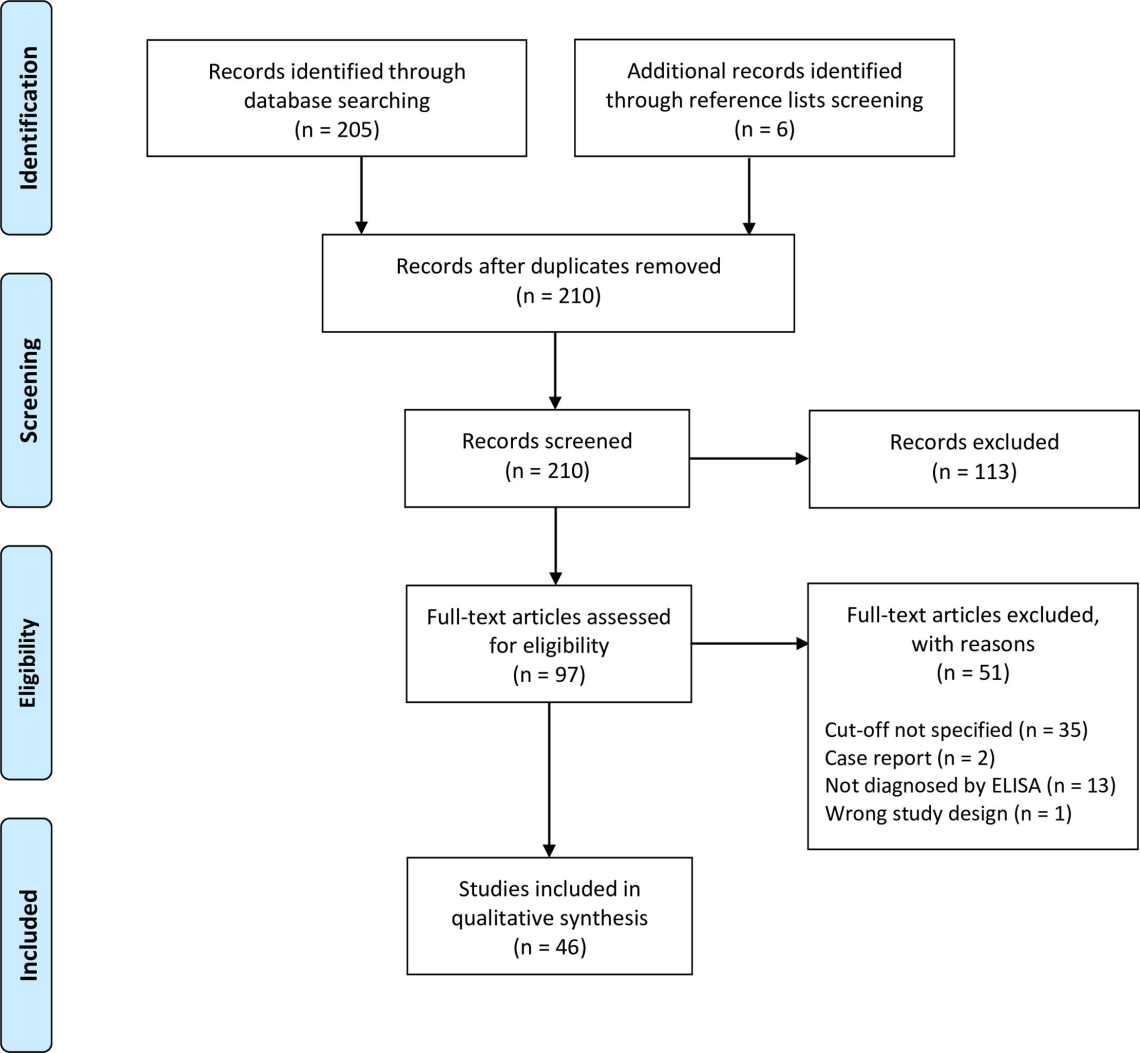
The Preferred Reporting Items for Systematic Reviews and Meta-Analyses (PRISMA) flow diagram.

Of the total of 46 studies included in this review (Fig 1), 24 (52.2%, 24/46) were observational studies and the remaining 22 (47.8%, 22/46) were diagnostic accuracy studies (Tables 1–3). Eighteen (81.8%, 18/22) of the diagnostic accuracy study tested the accuracy of ELISA against reference assays, while the remaining four (18.2%, 4/22) used ELISA as the reference assay.

**Table 1:** Summary of ELISA diagnostic accuracy studies.

Location	Year	Status	Antigen	Cut-off selection rationale	Isotype	Cut-off (OD)^a^	Reference test	Sample size	Study
Australia, Thailand	Not stated	In house	Karp and Gilliam r56-kDa Karp	ROC analysis Selected arbitrarily (for IgM and IgG in-combination ELISA)	IgM	Native Karp—0.4 Native Gilliam—0.2 r56 Karp—unclear In combination—0.45	IIP IgG titre ≥1:1,600, IgM ≥1:400	148	Land et al, 2000 [18]
					IgG	r56 Karp—0.5 In combination—0.9			
US soldiers (prior to deployment), Thailand	1986 1991–1992	In-house (NMRC)	Karp, Kato and Gilliam	Mean OD + 2SD ROC analysis	IgM	US Mean OD + 2SD—0.28	IIP IgG titre >1:1,600, IgM >1:400	373	Suwanabun et al, 1997 [19]
					IgG	US Mean OD +2SD—0.1			
						Thai Mean OD +2SD—0.42 ROC curve—0.8–1.3			
Thailand	1994–1995	In-house (NMRC)	Truncated r56-kDa Karp (from New Guinea)	Mean OD + 2SD	IgM	0.064 (1:400)	IIP with different cut-off titres	202	Ching et al, 1998 [22]
					IgG	0.11 (1:400)			
	1991–1992 1993–1999	In-house (NMRC)	Karp, Kato and Gilliam r56-kDa Karp	Mean OD + 2SD	IgM, IgG	Not stated	IIP IgG titre >1:1,600, IgM >1:400	430	Coleman et al, 2002 [31]
	2006–2007	Commercial (InBios)	r56-kDa	Mean OD + 3SD ROC analysis	IgM	0.6	IFA >1:400	152	Blacksell et al, 2015 [12]
						0.5	IFA>1:1,600		
						0.4–0.5	IFA 4-fold rise		
						0.4–0.5	IFA admission sample ≥ 1:3,200 or 4-fold rise to ≥1:3,200 in convalescent sample		
						0.4–0.5	Isolation		
						0.4	PCR		
						0.2–0.3	STIC (isolation, IFA admission ≥1:12,800, 4-fold rise, 2/3 positive PCR assays)		
						0.357	STIC (Mean OD + 3SD)		
						0.5	Based on all reference modalities		
	2007–2008	In-house (NMRC)	Karp, Kato and Gilliam	Bayesian LCM	IgM	1.474 (1:400 dilution)	PCR (2/3 assays), eschar, IFA admission titre ≥1:3,200/IFA admission ≥1:3,200 or 4-fold rise to ≥1:3,200 in convalescent-phase	135	Blacksell et al, 2016 [23]
	2010–2013	In-house (NMRC)	r56-kDa Karp and TA763, Kato, and Gilliam	Mean OD + SD (99% CI) ROC analysis	IgM	Mean OD + SD—0.320 ROC curve—0.360	IgG or IgM ≥400 (IFA) or 4-fold increase in IgG or IgM titre (IFA); or PCR positive	248	Chao et al, 2017 [24]
					IgG	Mean OD + SD—0.816 ROC curve—1.305			
India	2011–2012	Commercial (InBios)	r56-kDa	Not stated	IgM	0.5	IFA	1564	Mørch et al, 2017 [32]
	2011–2013	Commercial (InBios)	r56-kDa	ROC analysis	IgM	0.41	Unclear	145	Patricia et al, 2017 [25]
	2012–2013	Commercial (InBios)	r56-kDa	Recommendations from InBios kit protocol^b^	IgM	1.0	Micro-IFA—≥1:128	546	Koraluru et al, 2015 [14]
	2013–2015	Commercial (InBios)	r56-kDa	Mean OD + 3SD ROC analysis	IgM	Mean OD + 3SD—0.89 ROC curve—0.87	Response to antibiotic treatment within 48hr; and PCR or eschar	298	Gupta et al, 2016 [26]
	2013–2015	Commercial (InBios)	r56-kDa	Mean OD + 3SD	IgM	0.89	IFA >1:64	256	Gupta et al, 2017 [33]
	2012–2013	Commercial (InBios)	r56-kDa	Mean OD + 3SD	IgM, IgG	Not stated	ELISA was used as the reference test	45	Stephen et al, 2015 [34]
	2013–2014	Commercial (InBios)	r56-kDa	Mean OD + 3SD	IgM, IgG	Not stated	IFA IgM ≥ 1:10, IgG ≥ 1:40	87	Kim et al, 2016 [35]
	2013–2014	Commercial (InBios)	r56-kDa	Mean OD + 3SD	IgM, IgG	Not stated	ELISA was used as the reference test	127	Stephen et al, 2016 [36]
	2015–2016	Commercial (InBios)	r56-kDa	Mean OD + 3SD	IgM	0.56	ELISA was used as the reference test	240	Anitharaj et al, 2016 [37]
	Unclear	Commercial (InBios)	r56-kDa	Not stated	IgM	0.5	ELISA and eschar presence were used as the reference test	24	Janardhanan et al, 2014 [20]
Korea	1988–1991	In-house	r56-kDa Boryong	Mean OD + 3SD	IgM	~0.1	IFA seroconversion or 4-fold rise	170	Kim et al, 1993 [38]
	1997	In-house	r56-kDa Boryong	Mean OD + 3SD	IgM	0.2	IFA ≥ 1:80	176	Jang et al, 2003 [39]
	1999–2000	In-house	Chimeric r56-kDa 21-kDa Boryong 56-kDa Kangwon 87–61	Compared patients and negative controls	IgM, IgG	0.2	IFA seroconversion or 4-fold rise IgM IFA ≥ 1:10 IgG IFA ≥ 1:40	Unclear	Kim et al, 2013 [15]
Japan	2000–2012	In-house	Kato, Karp, Gilliam, Kuroki, and Kawasaki	Mean OD + 2, 3, and 4 SD	IgM, IgG	Mean + 3 SD (0.1789 for IgM and 0.2121 for IgG) and/or >4-fold rise of ELISA antibody titres for paired sera	Micro-IFA >1:80 and/or ≥4-fold rise for paired samples	49	Ogawa et al, 2017 [21]
China	Unclear	In-house	Truncated r56-kDa Ptan	Mean OD + 2SD	IgG IgM	0.16 (1:400) 0.12 (1:400)	Unclear	56	Cao et al, 2007 [40]

^a^ All cut-offs are for a 1:100 dilution, unless stated otherwise

^b^ InBios kits generally recommend a cut-off of the mean OD of non-scrub typhus serum samples + 3SD

**Table 2:** Summary of observational studies with a diagnostic accuracy component using InBios ELISAs.

Location	Sample collection time	Cut-off selection rationale	Isotype	Cut-off (OD)	Sample size	Study
India	2005–2010	Not stated	IgM	0.5	623	Varghese et al, 2014 [41]
	2009–2010	Not stated	IgM	1.0	259	Attur et al, 2013 [42]
	2009–2010	Not stated	IgM	0.5	154	Varghese et al, 2013 [43]
	2009–2011	Mean OD + 3SD	IgM	0.5	191	Astrup et al, 2014 [44]
	2010–2012	Mean OD + 2SD	IgM	0.6	167	Kalal et al, 2016 [45]
			IgG	0.37		
	2010–2012	Not stated	IgM	0.5	263	Varghese et al, 2015 [46]
	2011–2012	Not stated	IgM	0.5	42	Sengupta et al, 2014 [47]
	2012–2013	“As used in other studies”	IgM	0.5	284	Bhargava et al, 2016 [48]
	2013	Not stated	IgM, IgG	0.5	100	Sengupta et al, 2015 [27]
	2013–2014	Not stated	IgM	0.5	239	Sood et al, 2016 [49]
	2013–2014	Mean OD + 3SD	IgM	0.5	113	Usha et al, 2015 [50]
	2012–2015	Mean OD + 3SD	IgM	0.5	482	Roopa et al, 2015 [51]
	Unclear	Based on the mean of the ‘mixture distribution’	IgM	0.8	721	Trowbridge et al, 2017 [52]
			IgG	1.8		
China	2012–2014	Not stated	IgM	0.3	42	De et al, 2015 [53]
			IgG	0.5		
	2013–2014	Not stated	IgM, IgG	0.5	402	Hu et al, 2015 [28]
	2014–2016	Mean OD + 3SD	IgM	0.5	135	Chen et al, 2017 [54]
Malaysia	2007–2010	Mean OD + 3SD	IgG	Not stated	300	Tay et al, 2013 [55]
Sri Lanka	2012–2013	Mean OD + 3SD	IgM, IgG	Not stated	64	Pradeepan et al, 2014 [56]
Nepal	2015	Based on controls	IgM	0.5	434	Upadhyay et al, 2016 [57]

**Table 3:** Summary of observational studies using NMRC in-house ELISAs.

Location	Sample collection time	Cut-off selection rationale	Antigen	Isotype	Cut-off (OD)	Sample size	Study
Korea (US military)	1990–1995	Not stated	Karp, Kato and Gilliam	IgG	Initial screen– 0.5 (1:100) Second screen—Net total absorbance ≥1.0 Active infection—≥ 4-fold increase	9303	Jiang et al, 2015 [17]
Japan (US military)	2000 2001	Not stated	r56-kDa Karp, Kato, Gilliam Karp, Kato, Gilliam r56-kDa Karp ELISA	IgG	Titre >mean + 3SD or the titre that had an absorbance of at least 0.2 (whichever was greater). Confirmation—convalescent titter of >100 and a total absorbance >1	64	Jiang et al, 2003 [16]
Bangladesh	2010	Citing previous study	Karp and Gilliam	IgM	Net total absorbance ≥0.2 or ≥1.0 if there is no consensus	1250	Maude et al, 2014 [58]
Peru	2013	Not stated	Karp, Kato and Gilliam	IgG	Initial screen– 0.5 (1:100) Second screen—Net total absorbance ≥1.0 (1:100, 1:400, 1:1600, 1:6400) Active infection—≥ 4-fold increase, and minimum of 1:400 in convalescent sample	1124	Kocher et al, 2017 [59]
India	2013–2015	Not stated	Karp, Kato and Gilliam	IgG	Initial screen– 0.5 (1:100) Second screen—Net total absorbance ≥1.0 Active infection—≥ 4-fold increase	1265	Khan et al, 2016 [60]

#### Patient and geographic details

Study year of included articles varying from 1986 to 2016 (Tables 1–3). The total samples analyzed was 23,498, however one study did not provide the number of samples [15]. Geographically, the majority of the ELISA studies were conducted on serum samples from India (52.2%, 24/46), followed by Thailand (15.2%, 7/46), China (8.7%, 4/46) and Korea (8.7%, 4/46). The remaining study populations were recruited from Japan, the United States (US), Australia, Malaysia, Bangladesh, Sri Lanka, Peru and Nepal (Tables 1–3). Two studies investigated deployed US soldiers (one in Korea, and one in Japan) [16, 17]. Two studies investigated serum samples from two different countries [18, 19], and two studies did not specify a location [20].

### ELISA methodology

#### Source

The commercial ELISA kits manufactured by InBios (InBios International Inc., Seattle WA, USA) (referred to as InBios ELISAs) were the most numerous–being used in 30 studies (65.2%, 30/46) with the remainder being in-house assays. Ten studies (21.7%, 10/46) used ELISA methods developed by the US Naval Medical Research Centre (NMRC) (Tables 1 and 3).

#### Antigenic composition

There was a wide variety of native and recombinant antigens used in the ELISAs examined. The 30 InBios ELISAs identified in the study used a pool of recombinant 56-kDa proteins from Karp, Kato, Gilliam, and TA716 strains (r56-kDa) (Tables 1 and 2). Seven studies (15.2%, 7/46) used in-house ELISAs developed by NMRC, that employed whole-cell native antigens from Karp, Kato, Gilliam strains (Tables 1 and 3). While one study (2.2%, 1/46) used only Karp and Gilliam strain for its NMRC-developed in-house ELISAs (Table 3). There were two NMRC studies (4.3%, 2/46) that used recombinant Karp, Kato, Gilliam and TA763 strain or Karp alone antigens and two studies (4.3%, 2/46) with a combination of both whole cell and recombinant antigens. One study (2.2%, 1/46) used whole-cell Karp, Kato, Gilliam, and adding Kuroki and Kawasaki to the pool [21]. One study (2.2%, 1/46) employed combination of chimeric r56-kDa of Karp, Kato, and Gilliam strain, 21-kDa Boryong, and 56-kDa Kangwon 87–61 strain proteins as antigens. Remaining in-house ELISAs used variations of Karp, Kato, Gilliam, Boryong, and Ptan strains antigens (Tables 1 and 3).

### Diagnostic accuracy studies: Cut-offs used and methodology for selecting cut-offs

#### Diagnostic cut-offs

Eighteen studies (81.8%, 18/22) stated diagnostic cut-offs with considerable variation noted between the cut-offs (Table 1). Diagnostic cut-offs for IgM ranged from 0.064 [22] to 1.474 [23] OD (both 1:400 sample dilution) and IgG cut-offs ranged from 0.11 [22] to 1.305 [24] OD (both 1:100 sample dilution) which were all from Thai studies (Table 1). Variation was also apparent in cut offs selected for Indian studies, despite the exclusive use of the InBios ELISA where the IgM cut-offs ranged from 0.41 [25] to 1.0 [14] OD (Table 1).

Ten (43.5%, 10/22) studies investigated both IgG and IgM. Four of them (40.0%, 4/10) determined higher cut-off values for IgG than IgM. In two cases, the same cut-off was applied to both isotypes. For example, Kim *et al* calculated a cut-off of 0.2 OD for both isotypes despite using different reference standard cut-offs (Table 1) [15].

#### Methodology for selecting cut-offs

Using the reference comparator result to derive a diagnostic cut-off, six out of 22 diagnostic studies (27.3%, 6/22) performed Receiver Operator Characteristic (ROC) analysis, and one (4.5%, 1/22) used Bayesian latent class modelling (LCM).

Fifteen (83.3%, 15/18) diagnostic studies that determined ELISAs accuracy used IFA or IIP as the reference test. IFA/micro-IFA were used in 11 studies (61.1%, 1/18), with diagnostic cut-off titers ranging from 1:10 to 1:12,800 for IgM (Table 1). Five of these studies (45.5%, 5/11) also used a 4-fold rise between paired samples as a seropositivity criteria in addition to a defined cut-off titer. There were four (22.2%, 4/18) studies that used the indirect immunoperoxidase (IIP) assay as reference test. Two studies that assessed ELISA accuracy did not clearly mention the reference assay. Other reference modalities used include PCR, presence of eschar, *in vitro* isolation, and response to antibiotic treatment [12, 23, 24, 26]. In one study (4.5%, 1/22), a combination of the above was used in the form of scrub typhus infection criteria (STIC) composite [12].

The most commonly used method to determine a diagnostic cut-off was the addition of standard deviations (SD) to the mean OD of negative controls (72.7%, 16/22). There were four studies adding 2 SD, nine diagnostic studies adding 3 SD to the mean OD and one study calculated mean OD + 2, 3, and 4 SD (Table 1).

Variation in derived IgG cut-offs were noted within two studies that had each used both ROC curves and the mean OD to determine a cut-off for the same population [19, 24]. IgM cut-offs remained roughly the same when applying different methods (ROC curves and mean OD) in three studies [12, 24, 26]. The remaining 16 studies either gave unclear information on the method used, unjustified methods to determine seropositivity (four studies), arbitrarily selected cut-offs (one study), or cut-offs from unpublished data (one study).

### Observational studies: Cut-offs used and methodology for selecting cut-offs

Out of a total of 24 observational studies, seven (29.2%, 7/24) stated the method to determine the diagnostic cut-off and four (16.7%, 4/24) studies were unclear about how they derived the cut-offs stating “as used in other studies” or similar wording (Tables 2 and 3). Of the remaining studies, 13 (54.2%, 13/24) provided no clear explanation as to how the cut-off was selected, however 0.5 OD was used for IgM and/or IgG diagnosis for 11 (45.8%, 11/24) of these studies (Tables 2 and 3). Of the 19 observational studies using the InBios ELISA, seven (36.8%, 7/19) (Table 2) obtained local controls to determine a region-specific cut-off using the mean + 2 or 3 SD method. In the case of the NMRC in-house ELISA studies, the majority of studies (80.0%, 4/5) (Table 3), instead of calculating a single cut-off, patients were diagnosed with scrub typhus if they passed two criteria: 1) IgM OD ≥0.5 at a 1:100 dilution, and 2) a summed total OD of ≥1.0 of 4 sequential 4-fold dilutions. i.e., 1:100, 1:400, 1:1,600, 1:6,400) (Table 3).

## Discussion

The application of appropriate diagnostic cut-offs is important for timely scrub typhus patient management using appropriate antibiotic therapy and to prevent complications leading to significant detrimental effect. This review has determined that there was a significant lack of consensus regarding methodologies, application and diagnostic cut-offs for ELISAs used for the diagnosis of scrub typhus infections. However, the reasons are complex and require further investigation.

Approximately half of the observational studies provided no or insufficient justification for the OD cut-offs, and two studies did not specify the cut-off they used. Although the 0.5 OD cut-off was used commonly in InBios ELISAs studies and used by the Indian Council of Medical Research (ICMR), this is probably an appropriate estimation for certain parts of India with limited application in other geographic locations. This cut-off should be applied only in regions where it has been validated by testing samples from healthy controls to determine the level of background immunity in the normal population. In some cases, it is difficult to select a cut-off as demonstrated by Blacksell *et al*, where optimal OD cut-offs ranged from 0.2–0.6 OD depending on the reference standards used [12]. Several studies used the same cut-off for IgG and IgM, despite the differences in immunity dynamics of the different antibody isotypes–this should be taken into consideration when interpreting results of such tests [15, 24, 27, 28], as generally, upon infection a spike in IgM is seen, followed by increased levels of IgG, which also confers long-term protection.

There was a lack of uniformity of approach regarding the diagnostic accuracy studies to determine appropriate ELISA cut-offs for various geographic locations. The reference methodologies varied from Bayesian LCM using composite scrub typhus infection criteria (STIC), IFA, through to mean + SD in healthy controls. In most cases, there was no clear justification for the reference test cut-offs employed, and it is likely that some of these cut-offs were not appropriate for the location in which they were being used. For example, while an IFA cut-off of 1:400 is often set in Thailand, it has been suggested to have a high false-positivity rate [29]. Subsequently, the diagnostic accuracy of composites such STIC have also been suggested to overestimate scrub typhus positivity compared with index [30]. Bayesian LCM is being increasingly used to determine true diagnostic accuracies, as they do not assume any reference diagnostic test is perfect [30]. A recent study calculated–using this method–an admission IgM IFA cut-off of ≥1:3,200 or at least a 4-fold rise to ≥1: 3,200 in the convalescent-phase sample to provide the highest accuracy [29]. Only one study in this review used Bayesian LCM; combining IFA, PCR, eschar and culture results as reference standards to interpret ELISA results [23]. Given that the reference standards all have different accuracies, using a composite in a Bayesian approach helps to eliminate bias. Other studies used different approaches that may compromise accuracy. For example, in one study, a response to an unnamed antibiotic, along with positivity by either PCR or presence of an eschar, was used as the diagnostic criteria [26]. Generally, doxycycline is prescribed to treat scrub typhus, however since it is a broad-spectrum antibiotic and also used to treat leptospirosis and murine typhus, a response to treatment may not point specifically to scrub typhus as the cause of illness.

A number of factors may have an influence on the diagnostic accuracy of ELISAs including antigenic composition and sample population. Differences in ELISA methodologies were observed where studies used local antigenic strains or incorporated these into pooled Karp, Kato and Gilliam antigens to supposedly increase the accuracy of the test. In general, higher ODs were obtained when using homologous antigens, therefore variation in cut-offs were likely to be seen depending on the antigen being used and the locally circulating strains. In India, the use of the InBios ELISAs (which used Karp, Kato, Gilliam and TA716 strain antigens) was widely implemented, providing a more standardized means of diagnosing scrub typhus. Jiang et al demonstrated a trivalent r56-kDa protein to be superior to both monovalent r56-kDa Karp and whole-cell Karp, Kato and Gillam ELISAs [16]. The antigens used for deployed soldiers or travelers need to be carefully considered, and results need to be interpreted with caution, given their background immunity is likely to differ significantly from those living in endemic areas. Nevertheless, standardized, region-specific antigen preparations should be used in ELISAs, taking into consideration the circulating strains.

Regarding study populations, the use of samples from diseased or normal subjects as well as the geographic origin of the subjects can affect the derived diagnostic cut-off. In one study, serum samples were collected from Australia and Thailand, but it was unclear to which population the cut-off was applied, or whether the cut-off was calculated using results from both the populations despite differences in endemicity [18].

In addition to a lack of ELISA methodology standardization there was also lack of consensus in what is considered as the gold standard reference assay to determine diagnostic cut-offs. The absence of standardized methods and appropriate cut-offs has implications for seroepidemiology and clinical studies, as well as clinical decision making. On one hand, lower cut-off would result in false positives results risking unnecessary treatment and increasing probability of antimicrobial resistance. On the other hand, higher cut-off would result in false negative results risking cases to be missed.

This review has several limitations. First, it only investigated studies published in English, which may limit literature retrieval. Second, only one author performed the article selection and data extraction, however, any unclear data was discussed amongst the authors in order to limit bias. Lastly, the ELISA protocol was not examined as a factor. This needs to be considered when interpreting results, as differences in protocol (e.g. the amount of antigen used in plate coating) can influence the sensitivity and specificity of ELISA tests, that in turn influence the selection of optimal cut-offs. To limit the heterogeneity caused by different ELISA protocol, variations of the conventional ELISA were excluded from the review, and the InBios ELISA studies were grouped together in the analysis.

Further research will need to be conducted to determine local levels of background immunity, as well as to identify circulating strains, in order to make informed decisions for a region-specific, standardized ELISA methodology and cut-off.

## Supporting information

S1 Checklist PRISMA checklist. (DOC)

S1 Record PROSPERO record. (PDF)
