# Supplementary material for: The validity of diagnostic cut-offs for commercial and in-house scrub typhus IgM and IgG ELISAs: A review of the evidence
Source: PLoS Negl Trop Dis. 2019 Feb 4;13(2):e0007158. doi: 10.1371/journal.pntd.0007158 (PMC6382213; doi:10.1371/journal.pntd.0007158)
Supplement: S1 Record — (PDF) [file pntd.0007158.s002.pdf]

NHS
National Institute for Health Research

The validity of diagnostic cut-offs for commercial and in-house scrub typhus IgM and IgG ELISAs: a review of the evidence

Kartika Saraswati, Meghna Phanichkrivalkosil, Nicholas Day, Stuart Blacksell

Citation

Kartika Saraswati, Meghna Phanichkrivalkosil, Nicholas Day, Stuart Blacksell. The validity of diagnostic cut-offs for commercial and in-house scrub typhus IgM and IgG ELISAs: a review of the evidence. PROSPERO 2017 CRD42017078596 Available from:
http://www.crd.york.ac.uk/PROSPERO/display_record.php?ID=CRD42017078596

Review question

This review aims to summarize (1) the differences in ELISA methodologies, (2) the OD cut-offs used for diagnosing scrub typhus in research, and (3) the rationale behind the selection of certain OD cut-offs for scrub typhus diagnosis in previously published diagnostic accuracy studies and observational studies.

Searches

The PubMed database will be searched, using the following search terms: “scrub typhus,” “tsutsugamushi”, “immunoassay”, and “ELISA”.
The search will be restricted to articles published in English only, up to 16th October 2017.

Types of study to be included

Inclusion criteria: observational and diagnostic accuracy studies

Exclusion criteria: co-infection studies, reviews, case studies, and studies using variations of the conventional ELISA (eg dot ELISA)

Condition or domain being studied

Scrub typhus and ELISAs.
Scrub typhus is an acute febrile illness caused by Orientia tsutsugamushi which occurs mainly in the rural areas of Southeast Asia. ELISAs are often used to detect antibodies, but substantial variation exists in the 'cut-offs' used to diagnose patients.

Participants/population

Inclusion: patients with undifferentiated febrile illness.
Exclusion: patients with co-infections.

Intervention(s), exposure(s)

Designated cut-offs in ELISAs for the detection of antibodies against Orientia tsutsugamushi (the causative agent of scrub typhus).

Comparator(s)/control

Diagnosis using ELISAs compared with diagnosis by other reference methods, for example IFA, IIP, PCR, eschar, and STIC.

Context

Main outcome(s)

To assess the variation in cut-offs used for scrub typhus ELISAs, especially within endemic areas.

Timing and effect measures

Optical density (OD) value

Additional outcome(s)

To explore the reasons for the variations in cut-offs, such as the differences in ELISA methodologies employed (e.g. the use of different antigenic strains, the reference tests used, the positivity criteria used, etc.)

Data extraction (selection and coding)

The titles and abstracts retrieved in the searches will be screened for potential eligibility, after which the full texts of these will be assessed for inclusion/exclusion. A manual screening of the reference lists of relevant articles will also be carried out.

Data will then be extracted from the studies selected for inclusion, as follows:

Citation information;

ELISA brand/in-house;

Cut-off;

IgM/IgG;

Geographic location;

Methodology (for determining the cut-off);

Antigenic strain;

Reference test used;

Sample size.

Risk of bias (quality) assessment

No specific quality assessment will be performed. However, inconsistencies in the reporting relating to variables extracted will be noted.

Strategy for data synthesis

A narrative synthesis of the findings will be provided, structured around the differences in ELISA methodologies, and the methodologies used to determine the cut-offs used.

Analysis of subgroups or subsets

A subgroup analysis for OD cut-offs according to geographic location, or IgG/IgM may be conducted.

Contact details for further information

Meghna Phanichkrivalkosil
meghnakosil96@gmail.com

Organisational affiliation of the review

Mahidol-Oxford Tropical Medicine Research Unit
http://www.tropmedres.ac/home

Review team members and their organisational affiliations

Dr Kartika Saraswati. Wellcome Trust-Mahidol University-Oxford Tropical Medicine Programme, Faculty of Tropical Medicine, Mahidol University, Bangkok, Thailand. Centre for Tropical Medicine and Global Health, Nuffield Department of Medicine, Nuffield Department of Medicine Research Building, University of Oxford, Oxford, United Kingdom. Eijkman-Oxford Clinical Research Unit, Eijkman Institute for Molecular Biology, Jakarta, Indonesia
Ms Meghna Phanichkrivalkosil. Wellcome Trust-Mahidol University-Oxford Tropical Medicine Programme, Faculty of Tropical Medicine, Mahidol University, Bangkok, Thailand.

Dr Nicholas Day. Wellcome Trust-Mahidol University-Oxford Tropical Medicine Programme, Faculty of Tropical Medicine, Mahidol University, Bangkok, Thailand. 2Centre for Tropical Medicine and Global Health, Nuffield Department of Medicine, Nuffield Department of Medicine Research Building, University of Oxford, Oxford, United Kingdom.
Dr Stuart Blacksell. Wellcome Trust-Mahidol University-Oxford Tropical Medicine Programme, Faculty of Tropical Medicine, Mahidol University, Bangkok, Thailand. 2Centre for Tropical Medicine and Global Health, Nuffield Department of Medicine, Nuffield Department of Medicine Research Building, University of Oxford, Oxford, United Kingdom.

Anticipated or actual start date

04 September 2017

Anticipated completion date

29 September 2018

Funding sources/sponsors

Nicholas Day, Kartika Saraswati, and Stuart Blacksell are funded by the Wellcome Trust of Great Britain

Conflicts of interest

None known

Language

English

Country

Thailand

Stage of review

Review_Completed_not_published

Subject index terms status

Subject indexing assigned by CRD

Subject index terms

Antibodies, Bacterial; Bacterial Infections; Enzyme-Linked Immunosorbent Assay; Humans; Molecular Diagnostic Techniques; Orientia tsutsugamushi; Scrub Typhus

Date of registration in PROSPERO

28 November 2017

Date of publication of this version

07 November 2018

Details of any existing review of the same topic by the same authors

Stage of review at time of this submission

Stage	Started	Completed
Preliminary searches	Yes	Yes
Piloting of the study selection process	Yes	Yes
Formal screening of search results against eligibility criteria	Yes	Yes
Data extraction	Yes	Yes
Risk of bias (quality) assessment	Yes	Yes
Data analysis	Yes	Yes

Versions

01 January 1900
07 November 2018

PROSPERO
This information has been provided by the named contact for this review. CRD has accepted this information in good faith and registered the review in PROSPERO. CRD bears no responsibility or liability for the content of this registration record, any associated files or external websites.
